# Oleanolic Acid Exerts Osteoprotective Effects and Modulates Vitamin D Metabolism

**DOI:** 10.3390/nu10020247

**Published:** 2018-02-22

**Authors:** Sisi Cao, Xiao-Li Dong, Ming-Xian Ho, Wen-Xuan Yu, Ka-Chun Wong, Xin-Sheng Yao, Man-Sau Wong

**Affiliations:** 1 Department of Applied Biology and Chemical Technology, The Hong Kong Polytechnic University, Hung Hom, Kowloon, Hong Kong, China; sissi.cao@connect.polyu.hk (S.C.); bcxldong@polyu.edu.hk (X.-L.D.); mxianho@hotmail.com (M.-X.H.); wx.yu@polyu.edu.hk (W.-X.Y.); aguesses@yahoo.com.hk (K.-C.W.); 2 Shenzhen Key Laboratory of Food Biological Safety Control, The Hong Kong Polytechnic University Shenzhen Research Institute, Shenzhen 518057, China; 3 Institute of Traditional Chinese Medicine & Natural Products, College of Pharmacy, Jinan University, Guangzhou 510632, China; tyaoxs@jnu.edu.cn; 4 State Key Laboratory of Chinese Medicine and Molecular Pharmacology (Incubation), The Hong Kong Polytechnic University Shenzhen Research Institute, Shenzhen 518057, China

**Keywords:** oleanolic acid, ovariectomised, aging, osteoporosis, calcium, vitamin D

## Abstract

Oleanolic acid (OA) is a triterpenoid with reported bone anti-resorption activities. The present study aimed to characterize its bone protective effects in vivo and to study its effects on vitamin D metabolism, both in vivo and in vitro. OA significantly increased bone mineral density, improved micro-architectural properties, reduced urinary Ca excretion, increased 1,25(OH)_2_D_3_ and renal CYP27B1 mRNA expression in mature C57BL/6 ovariectomised (OVX) mice. OA also improved bone properties, Ca balance, and exerted modulatory effects on renal CYP27B1 and CYP24A1 expressions in aged normal female Sprague–Dawley rats. In addition, OA significantly increased renal CYP27B1 mRNA and promoter activity, and suppressed CYP24A1 mRNA and protein expressions in human proximal tubule HKC-8 cells. OA exerted bone protective effects in mature OVX mice and aged female rats. This action on bone might be, at least in part, associated with its effects on Ca and vitamin D metabolism. The present findings suggest that OA is a potential drug candidate for the management of postmenopausal osteoporosis.

## 1. Introduction

Osteoporosis is a metabolic bone disease that increases fracture risk in the elderly and poses significant social and economic burden to the world’s population. The worldwide estimated osteoporosis-related fracture rate is around 40% in aged women and about 13% in men [1]. Aging [2] and estrogen deficiency [3] are the two major leading causes of osteoporosis. The age-related changes in calcium homeostasis may contribute to the chronic depletion of calcium from bone [4]. Indeed, the impaired intestinal calcium absorption during aging is believed to be the consequence of the age-related decline in renal 1,25(OH)_2_D_3_ production and intestinal vitamin D receptor (VDR) expression that lead to reduced 1,25(OH)_2_D_3_ sensitivity [5]. The reduction of estrogen level during menopausal transition contributes to rapid bone loss in women, with an average decline of 10% bone mineral density (BMD) in the five years around menopause [3]. The estrogen deficiency-induced bone loss in postmenopausal women is complicated by the age-induced abnormalities in the vitamin D endocrine system [6]. Vitamin D deficiency-induced secondary hyperparathyroidism leads to an increase in the rate of bone loss [2]. Calcium and vitamin D supplements are commonly used by adults for the management of bone health [7]. However, concerns have been raised regarding the potential increase in the risk of cardiovascular events by the use of Ca supplements in healthy postmenopausal women [8,9]. In addition, the safety data concerning long term use of high-dose vitamin D3 supplementation in elderly, especially those with compromised renal function, is limited [10]. Moreover, recent cohort studies and systematic reviews suggested a weak association between increased dietary intake of calcium or calcium and/or vitamin D supplements and a reduced fracture risk in the older population [11,12,13,14]. Thus, alternative approaches for the improvement of calcium balance and long-term management of bone health are needed.

**Figure 1:**
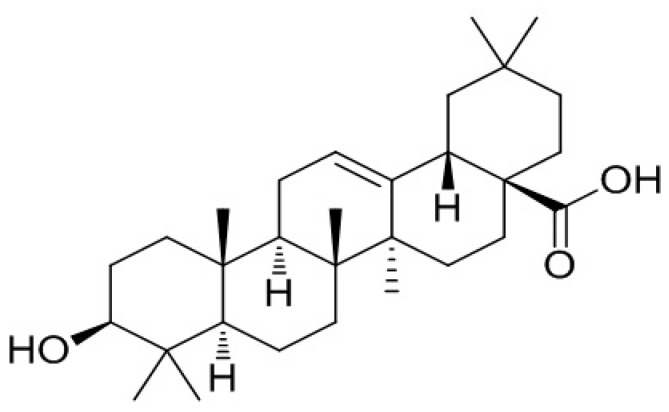
Chemical structure of oleanolic acid.

Oleanolic acid (3β-hydroxyolean-12-en-28-oic acid) (Figure 1) is a bone protective pentacyclic triterpenoid compound [15] that has been identified in over 1620 dietary plants and medicinal herbs [16]. Oleanolic acid (OA) glucosides and its derivatives have been shown to reduce the formation of osteoclast-like multinucleated cells in a primary co-culture system [17,18,19]. Oral administration of 0.1–10 mg/kg/day quinoxaline derivative of OA for 5 weeks significantly improved BMD in ovariectomised (OVX) mice [20]. Similarly, oral feeding with 20 mg/kg/day OA for 3 months was shown to protect against bone loss in OVX rats, and OA was also shown to induce osteoblastic differentiation of bone mesenchymal stem cells (MSCs) [21]. In addition, treatment of male mice with OA acetate for eight days effectively prevented lipopolysaccharide-induced inflammatory bone loss [22]. Mechanistic studies demonstrated that the inhibitory effects of OA on osteoclastogeneis were mediated by the receptor activator of NF-κB ligand (RANKL) signaling pathway in RAW264.7 cells [23], while the stimulatory effects of OA on human MSCs towards osteoprogenitor cells involved the inhibition of Notch signaling pathways [24].

Our group was the first to report that *Fructus Ligustri Lucidi* (FLL), an OA-rich Chinese herb, could regulate bone turnover markers and enhance calcium balance in mature OVX rats [25]. Indeed, OA is being used as the authentication marker [26] of FLL. Our subsequent studies showed that FLL significantly improved calcium balance and bone properties in both aged ovary-intact [27] and aged OVX rats [28,29]. The improvement of bone properties in rats by FLL appeared to be associated with its actions on the vitamin D–parathyroid hormone (PTH) axis [30,31]. Recent studies further reported that FLL exerted stimulatory effects on bone and calcium balance in growing male and female rats [32,33]. These studies clearly show that FLL exerts bone protective effects through regulating calcium and vitamin D metabolism in growing, mature, and aged animal models. Thus, it is of interest to determine whether OA, as the major bioactive identified in FLL, could also exert its bone protective effects via modulation of the calcium–vitamin D axis.

1α,25-dihydroxyvitamin D_3_ (1,25(OH)_2_D_3_) is essential for normal bone mineralization and calcium homeostasis in the body [34]. The circulating concentration of 1,25(OH)_2_D_3_ is tightly controlled by the biosynthetic enzyme, 25-hydroxyvitamin D 1-α-hydroxylase (CYP27B1), as well as the catalytic enzyme, 25-hydroxyvitamin D 24-hydroxylase (CYP24A1) [35]. CYP27B1 is primarily located in the proximal tubule of the nephron [36] and is tightly regulated by many hormonal factors, including parathyroid hormone (PTH) [37], 1,25(OH)_2_D_3_ itself [38], fibroblast growth factor 23 (FGF23) [39], as well as minerals, such as Ca and phosphate (P) ions [40,41]. CYP24A1 is induced by 1,25(OH)_2_D_3_ [42] and FGF-23 [43] and suppressed by PTH [42]. A previous study showed that FLL could increase circulating 1,25(OH)_2_D_3_ by inducing renal CYP27B1 activity in rat renal proximal tubule cells [30]. Thus, it is of interest to determine if OA could also increase circulating 1,25(OH)_2_D_3_ and alter vitamin D metabolism.

The present study was designed to test the hypothesis that OA could protect against estrogen deficiency- and age-induced bone loss via its actions involving the promotion of calcium absorption and regulation of vitamin D metabolism. The effects of OA on bone properties and calcium–vitamin D metabolism in both mature OVX mice and aged female rats as well as its effects on vitamin D metabolic enzymes in human proximal tubule (HKC-8) cells were determined. The results provide evidence of the osteoprotective effects of OA in OVX mice and aged rats.

## 2. Materials and Methods

### 2.1. Animal Study Design

#### 2.1.1. Experiment 1: Bone Protective Effect of OA in OVX Mouse Model

Forty-two four-month-old C57BL/6J mice were purchased from The Chinese University of Hong Kong. Mice were randomly subjected to either sham-operated or ovariectomy (OVX) and were orally administrated by daily gavage for 6 weeks, as follows: sham + vehicle (Sham, *n* = 9), OVX + vehicle (OVX, *n* = 9), OVX + 17β-oestradiol (E_2_, 200 μg/kg/day, *n* = 8), OVX + OAL (low dose of OA, 50 mg/kg/day, *n* = 8), and OVX + OAH (high dose of OA, 100 mg/kg/day, *n* = 8). The animals were pair-fed with 3 g, the minimum daily average food intake, phytoestrogen-free AIN-93M rodent diet (Research diets, New Brunswick, NJ, USA). The 17β-Oestradiol (E_2_) was from Sigma–Aldrich (Sigma, St. Louis, MO, USA) and OA (purity > 98%) was from Shanghai Winherb Medical Technology Co. (Shanghai, China). The body weights of the animals were monitored on a weekly basis. One day before sacrifice, mice were individually housed in metabolic cages for urine collection. The mice were then sacrificed by cardiac stick exsanguinations under anaesthesia. Serum was prepared in aliquots and stored at −80 °C for biochemical measurements. The uterine index was calculated from the wet weight of uterus over the body weight. The left tibia and intact lumbar vertebra were collected, with soft tissue cleared, wrapped in saline-soaked gauze and stored at −20 °C for micro-computed tomography (μCT) analysis.

#### 2.1.2. Experiment 2: Effect of OA on Bone and Calcium Balance in an Aged Female Rat Model

Twenty-four retired breeder Sprague–Dawley (SD) rats, aged nine-months-old, were purchased from Beijing Vital River Laboratories (Beijing, China). The retired breeders were raised to thirteen-months-old to establish the aged rat model. Before the treatment regimen, rats were pair-fed with a normal calcium diet (NCD, TD98005, 0.6% calcium, 0.65% phosphorus) for five days as acclimation. Aged female rats were randomly assigned to three groups (*n* = 8/group): control diets with oral administration of OA (25 mg/kg/day) or its vehicle by daily gavage, and a high calcium diet (HCD, 1.2% calcium, 0.65% phosphorus) served as a positive control group. All animals were pair-fed with 15 g/day of a control diet with water supply *ad libitum* for 12 weeks. Diets were purchased from Harlan Teklad (Madison, WI, USA). Before sacrifice, rats were individually placed into metabolic cages for 24 h urine and feces collection. Upon sacrifice, blood, duodenal mucosa, and cortex of the left kidney were collected. Serum in aliquots and tissue samples were stored at −80 °C for further analysis. The left tibia, femur, and the intact lumbar vertebra were collected with soft tissues cleaned, wrapped by phosphate buffered saline (PBS)-soaked gauze, and stored at −20 °C for micro-CT scanning and bone calcium content measurement.

All animals were housed in a room at 22 °C and provided with a 12 h light and dark cycle. All the experimental procedures were approved by the Animal Ethics Committee of The Hong Kong Polytechnic University (ASESC No.: 14-15/04-ABCT-R-STUDENT (mice study) and ASESC No.: 130602 (rat study)).

### 2.2. Biochemical Assays of Serum and Urine Samples

The concentrations of calcium and phosphorous in serum and urine samples were measured by standard colorimetric methods, using commercial kits, following the manufacturers’ instructions (STANBIO laboratory, Boerne, TX, USA). Mice serum osteocalcin (OCN) level was measured by a mouse osteocalcin ELISA kit (Alfa Aesar, Lancashire, UK). Mice urinary deoxypyridinoline (DPD) level was measured using a METRATM DPD EIA kit (Quidel Corporation, San Diego, CA, USA). Serum 1,25(OH)_2_D_3_ level was determined using a mouse 1,25-dihydroxyvitamin D_3_ (1,25(OH)_2_D_3_) ELISA kit (BlueGene Biotech, Shanghai, China) for OVX mice and a 1,25-(OH)_2_-Vitamin D ELISA Kit (Immundiagnostik AG, Bensheim, Germany) for aged rats. Rat serum PTH level was determined by Rat Bio Active Intact PTH ELISA Kit (Immutopics, Inc., San Clemente, CA, USA). Urinary calcium, phosphorous, and DPD were corrected by urinary creatinine (Cr) levels, which were determined by picric acid methods, using commercial kits (Zhongsheng Beikong Bio-technology and Science Inc., Beijing, China).

### 2.3. Microcomputed Tomography (μCT)

The left tibias, left femurs and fourth lumbar vertebrae (L4) of OVX mice and aged rats were scanned at voxel sizes of 10.5 μm^3^ and 21 μm^3^, respectively, by the μCT system (viva-CT40; Scanco Medical, Bassersdorf, Switzerland). Scans were performed at a medium resolution and using energy of 70 kVp, intensity of 114 μA, with an integration time of 300 ms. Distal femurs and proximal tibias were scanned in 100 slices for OVX mice and 210 slices for aged rats, from the metaphyseal growth plates. The volume of interest was contoured from 50 or 100 serials of slices (corresponding to a 0.525 mm region and 2.1 mm region), starting from the disappearance of the condyle, for evaluation. For the lumbar vertebra (L4), 100 slices for OVX mice and 150 slices centered in L4 for aged rats were scanned and evaluated. Morphometric parameters included bone mineral density (BMD, mg HA/cm^3^), bone volume/tissue volume (BV/TV, %), trabecular number (Tb.N, 1/mm), trabecular thickness (Tb.Th, mm), trabecular separation (Tb.Sp, mm), and connectivity density (Conn.D, 1/mm^3^) were calculated using a three-dimensional direct model with a constant threshold of 300.

### 2.4. Bone Calcium Content

The left tibia of both OVX mice and aged rats were first dried at 110 °C in muffle furnace for 12 h and then incinerated at 800 °C for 20 h. The ash weight was recorded. 100 mg of bone ash was weighed and dissolved in 2 mL of 37% HCl and diluted by Milli-Q water for atomization. The bone calcium content was determined by atomic absorption spectrophotometer (PerkinElmer, AAnalyst 100 Spectrometer, Norwalk, CT, USA).

### 2.5. Calcium Balance Study

The calcium content in 24 h fecal samples from aged rats was determined by the same method as for bone calcium, described previously [25]. The calcium absorption rate and calcium balance were calculated using the following equations: Ca absorption rate (%) = (Ca intake − fecal Ca)/Ca intake × 100; Ca balance = Ca intake − (urinary Ca + fecal Ca).

### 2.6. Cell Culture Study

Human proximal kidney tubule cells (HKC-8) were a kind gift from Dr. Racusen of Johns Hopkins University [44]. Cells were cultured in DMEM/F12 medium (Life Technologies, Carlsbad, CA, USA), supplemented with 1X penicillin/streptomycin and 5% Fetal Bovine Serum (FBS) (Life Technologies, Carlsbad, CA, USA). The cell culture was maintained on 100 mm culture dishes in the incubator, in a 5% CO_2_–95% air atmosphere, at 37 °C. Forskolin (FSK, #F6886, purity ≥ 98%, Sigma–Aldrich, St. Louis, MO, USA) and 1,25(OH)_2_D_3_ (1,25D, #D1530, purity ≥ 99%, Sigma–Aldrich, St. Louis, MO, USA) were applied as positive controls for determining CYP27B1 and CYP24A1 expressions, respectively. For assessment of promoter activities, mRNA, and protein expressions, the medium was changed to a chemically–defined, serum-free medium containing the following supplements for 24 h before drug treatment: insulin (5 μg/mL), transferrin (5 μg/L), Na_2_SeO_3_ (5 ng/mL), tri-iodothyronine (0.37 nmol/L), epidermal growth factor (2.5 ng/mL), and hydrocortisone (1 nmol/L).

### 2.7. Real-Time Polymerase Chain Reaction (PCR) Analysis

RNA from animal tissues and HKC-8 cells was isolated using TRIzol^®^ Reagent (Invitrogen, Carlsbad, CA, USA). Total RNA (4 μg) was reverse-transcribed by M-MLV Reverse Transcriptase (Invitrogen, Carlsbad, California, USA) following the manufacturer’s instructions. Real-time PCR was performed in a total of 20 μL of reaction mixture, containing 10 μL of SYBR Green Mastermix (Applied Biosystems, Carlsbad, CA, USA) and 0.5 μL of cDNA product by 7900HT Fast Real-Time PCR System (Applied Biosystems, Carlsbad, CA, USA) using the two-step program: initial denaturation at 95 °C for 10 min, 40 cycles of denaturation at 95 °C for 15 s and 60 °C for 1 min. The sequences of primers for target genes and the housekeeping gene glyceraldehyde-3-phosphate dehydrogenase (GAPDH) are listed in Table 1. The relative quantity of mRNA was calculated by fitting the Ct value to the standard curve using the SDS software package (Applied Biosystems, Carlsbad, CA, USA), and each gene expression was normalized using its own GAPDH expression level.

### 2.8. Western Blot Analysis

Cortexes of kidneys and HKC-8 cells were homogenized and lysed in Nonidet P-40 Lysis Buffer (20 mM Tris-HCl, pH 7.5; 150 mM NaCl, 1 mM MgCl_2_, 10% glycerol, 1% Nonidet P-40) supplemented with protease inhibitors: 1 mM PMSF, 2 μg/mL aprotinin, 2 μg/mL leupeptin, 10 mM NaF, and 1 mM sodium orthovanadate (Sigma, St. Louis, MO, USA). Protein concentrations were determined using the Bradford protein assay (Bio-Rad, Philadelphia, PA, USA). The proteins were separated by sodium dodecyl sulfate polyacrylamide gel electrophoresis (SDS-PAGE) and transblotted to polyvinylidene difluoride (PVDF) membranes (Immobilin-P, Millipore Corp., Bedford, MA, USA) and probed with the primary antibodies: rabbit anti-CYP27B1 (1:500, Santa Cruz Biotechnology, Santa Cruz, CA, USA), rabbit anti-CYP27B1 (1:500, Santa Cruz Biotechnology, Santa Cruz, CA, USA), or mouse anti-β actin (1:5000, Abcam, Cambridge, MA, USA), followed by IgG-HRP-conjugated secondary antibodies anti-rabbit (1:2000, Santa Cruz Biotechnology, Santa Cruz, CA, USA) or anti-mouse (1:3000, Cell Signaling Technology, Beverly, MA, USA). Membranes were incubated with enhanced chemiluminescence (ECL) substrate (ClarityTM Western ECL Substrate, Bio-Rad, Philadelphia, PA, USA) for 5 min. The bound antibodies were visualized and quantified by Lumi-Imager (Roche, Manheim, Germany). The signal intensity of the bands was presented as Biochemical Light Units (BLU).

### 2.9. Transient Transfection

HKC-8 cells were transiently transfected with CYP27- or CYP24-promoter construct. The CYP27-promoter construct, a reporter plasmid containing a full-length insert of 1576 bp CYP27B1 promoter region, was a kind gift from Dr. Farzana Perwad from University of California, San Francisco [45]. CYP24-promoter construct, a reporter plasmid containing a 300-bp vitamin D responsive region of the CYP24 promoter, was generated by the late Dr. Jack Omdahl [46] and was kindly provided by Dr. JoEllen Welsh from University at Albany-SUNY [47]. HKC-8 cells were co-transfected in serum-free medium with 0.4 μg of CYP27- or CYP24-promoter plasmid, together with 0.1 μg constitutive thymidine kinase promoter plasmid (pRL-TK, Promega, Madison, WI, USA) using FuGene HD transfection reagent (Promega). After 4 h of incubation, cells were treated with vehicle (0.1% ethanol), FSK (10^−5^ M), 1,25D (10^−8^ M) or OA (10^−8^–10^−5^ M) for 24 h. A dual luciferase reporter assay was conducted using reagents from Promega. The promoter activities of CYP27 or CYP24 were normalized to pRL-TK. Data were presented as relative luciferase units (RLU).

### 2.10. Statistical Analysis

All data obtained from the experiments were presented in the form of mean ± SEM (standard error of mean). The differences between groups of data were analyzed by GraphPad Prism Version 6.00 (GraphPad Software, La Jolla, CA, USA). The significances between different groups of means were evaluated by one-way analysis of variance (ANOVA). Post-test analysis of multiple comparisons was carried by Tukey’s test at a confidence level of 95%. *p*-values less than 0.05 were considered to be statistically significant.

## 3. Results

### 3.1. Effect of OA on Body Weight, Uterus Index and Biochemical Parameters in OVX Mice

Ovariectomy significantly increased the body weight of mice (*p* < 0.001 vs. sham), and treatment of mice with 17β-oestradiol (E2) and both dosages of OA significantly suppressed OVX-induced body weight (*p* < 0.001 vs. OVX, Table 2). Significant atrophy of the uterus was found in OVX mice, suggesting that the surgery was successful (*p* < 0.001 vs. sham). Unlike E2, OA treatment in low and high dose groups did not induce a uterus index in OVX mice (Table 2). Serum calcium, serum and urinary phosphorus levels were not altered by OVX or any intervention, while urinary calcium loss induced by OVX was suppressed in mice treated with E2 (*p* < 0.05 vs. OVX) and with OA (*p* < 0.001 vs. OVX, Table 2). In addition, serum 1,25(OH)_2_D_3_ levels were significantly increased in OVX mice treated with OA at a high dose (*p* < 0.05 vs. OVX, Table 2), but were not altered in other treatment groups. 

### 3.2. Effect of OA on Bone Markers, Bone Calcium Content and Bone Micro-Architecture in OVX Mice

Serum osteocalcin (OCN) and urinary deoxypyridinoline (DPD) levels were significantly increased in OVX mice (vs. Sham). Treatment of OVX mice with E2, but not OA, significantly suppressed OVX-induced serum OCN levels and urinary DPD levels (vs. OVX, Table 2). Bone ash weight and calcium content were not significantly altered in mice in response to ovariectomy. Treatment of OVX mice with E2 and OA at both dosages significantly increased bone ash weight and calcium content in the tibia (*p* < 0.05 vs. OVX, Table 2). As shown in Table 3, ovariectomy significantly reduced trabecular BMD in the proximal tibia, distal femur as well as in lumbar vertebra L4 in mice by 44%, 34%, and 21%, respectively (*p* < 0.001 vs. Sham). Treatment of OVX mice with E2 and OA at both dosages significantly increased trabecular BMD at all three bone sites. In addition, the OVX-induced bone micro-architecture deterioration in mice was effectively prevented by the administration of E2 (vs. OVX) and both dosages of OA treatments (vs. OVX), as revealed by the significant improvements in BV/TV and Conn.D, and decrease in Tb.Sp at all three sites of bone in OVX mice. Treatment of OVX mice with OA also increased Tb.N at the distal femur and L4, and increased Tb.Th at the proximal tibia and distal femur (Table 3).

### 3.3. Effect of OA on Renal CYP27B1 and CYP24A1 mRNA and Protein Expression in OVX Mice

**Figure 2:**
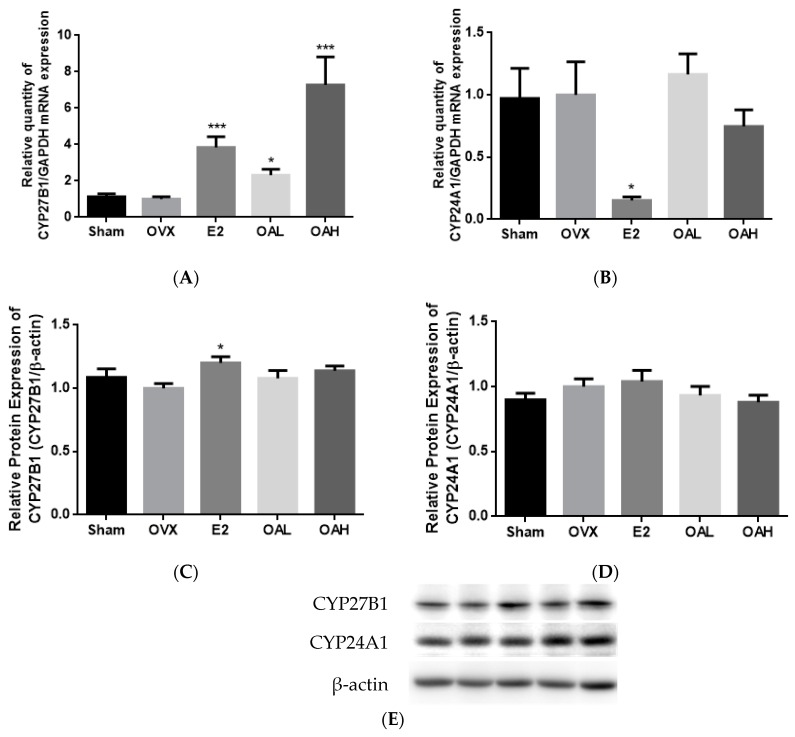

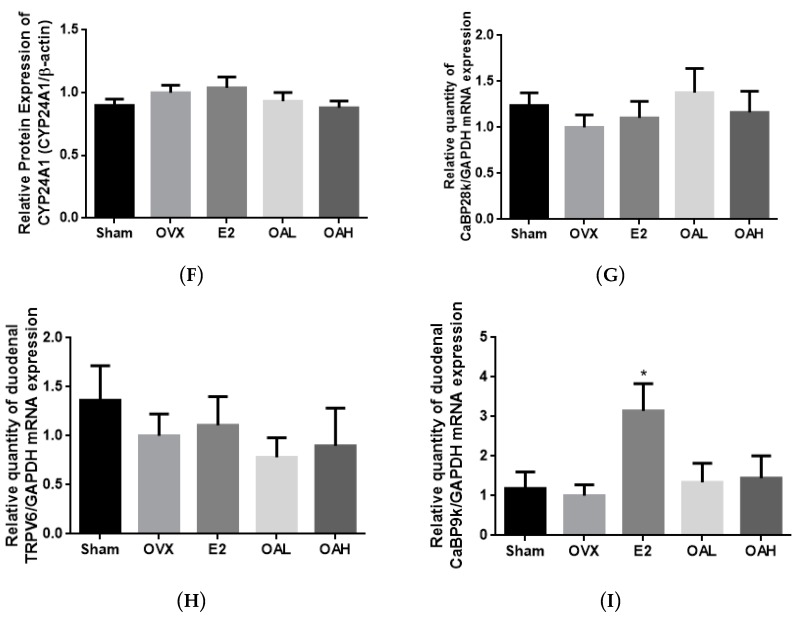
The effects of OA on renal and duodenal mRNA and protein expressions in OVX mice. The expression levels of (**A**) renal CYP27B1 mRNA; (**B**) renal CYP24A1 mRNA; (**C**,**E**) renal CYP27B1 protein; (**D**,**E**) renal CYP24A1 protein; (**F**) renal TRPV5 mRNA; (**G**) renal CaBP28k mRNA; (**H**) duodenal TRPV6 mRNA; and (**I**) duodenal CaBP9k mRNA in OVX mice were studied. Four-month-old ovariectomized (OVX) or sham-operated (Sham) C57BL/6J mice were pair-fed with phytoestrogen-free AIN-93M diet and treated with vehicle (Sham or OVX), E2 (200 μg/kg/day), OA low dose (OAL, 50 mg/kg/day) or OA high dose (OAH, 100 mg/kg/day) for 6 weeks. The mRNA expression level is presented as the ratio of target gene to GAPDH. The protein expression level is shown as the ratio of target protein to β-actin. Data are presented by mean ± SEM and analyzed by one-way ANOVA followed by Tukey’s multiple comparison tests. * *p* < 0.05 and *** *p* < 0.001 vs. OVX.

The regulatory effects of OA on renal vitamin D metabolic enzymes were then investigated. E2 and OA significantly induced renal CYP27B1 mRNA expression in OVX mice (*p* < 0.001 vs. OVX, Figure 2A). However, E2, but not OA, significantly stimulated renal CYP27B1 protein expression (*p* < 0.05 vs. OVX, Figure 2C,E) and suppressed renal CYP24A1 mRNA expression (*p* < 0.05 vs. OVX, Figure 2B). OA at a high dose tended to decrease renal CYP24A1 mRNA and protein expressions but the changes did not reach statistical significance (Figure 2B,D,E).

### 3.4. Effect of OA on Renal and Duodenal mRNA Expression for Calcium Transport in OVX Mice

To determine if the suppression of urinary calcium loss by OA treatment in mice might be associated with an increase in renal calcium re-absorption or increase in duodenal calcium absorption, the gene expressions of epithelial calcium channels, renal TRPV5 and duodenal TRPV6, as well as the calcium-binding proteins (CaBPs), renal calbindin D_28k_ (CaBP28k) and duodenal calbindin D_9k_ (CaBP9k) were studied. Both E2 and OA treatments were found to significantly induce the mRNA expression of renal calcium channel TRPV5 (*p* < 0.05 vs. OVX, Figure 2F) but did not alter renal CaBP28k (Figure 2G) or duodenal TRPV6 (Figure 2H) mRNA expression in OVX mice. E2, but not OA, increased duodenal CaBP9k mRNA expression in OVX mice (Figure 2I).

### 3.5. Effect of OA on Body Weight and Biochemical Parameters in Aged Rats

The body weight of aged rats remained unchanged during the study period. Neither a high calcium diet (HCD, 1.2% calcium), nor OA, altered serum calcium and phosphorous levels in aged rats. HCD treatment significantly decreased urinary phosphorous excretion (*p* < 0.001 vs. NCD) and serum 1,25(OH)_2_D_3_ levels (*p* < 0.001 vs. NCD) but did not alter urinary calcium and serum PTH levels in aged rats. In contrast, treatment of aged rats with OA did not change urinary phosphorous levels but effectively reduced urinary Ca/Cr levels in aged rats (*p* < 0.05 vs. NCD). However, OA treatment did not alter serum 1,25(OH)_2_D_3_ levels nor serum PTH levels in aged rats (Table 4).

### 3.6. Effect of OA on Calcium Balance in Aged Rats

Table 5 summarizes the results of calcium balance in aged rats following OA treatment. As expected, the HCD group had significant higher urinary (*p* < 0.001 vs. NCD) and fecal calcium loss (*p* < 0.01 vs. NCD) and enhanced calcium balance (*p* < 0.05 vs. NCD) in aged rats. Administration of OA to aged rats significantly enhanced net calcium balance by two-fold (*p* < 0.05 vs. NCD) to a comparable level with that of the HCD group.

### 3.7. Effect of OA on Bone Calcium Content and Bone Micro-Architecture in Aged Rats

OA significantly induced bone calcium content in the tibia of aged rats to a comparable level with that of the HCD group (*p* < 0.05 vs. NCD, Table 4), whereas, HCD, but not OA, significantly increased bone ash weight in aged rats (*p* < 0.01 vs. NCD, Table 4). The effect of HCD and OA on BMD and micro-architectural properties at the proximal tibia, distal femur and lumbar vertebra (L4) in aged rats are shown in Table 6. Bone loss in aged rats was rescued by treatment with a HCD and OA at all three bone sites measured (vs. NCD). OA significantly increased trabecular BMD at the proximal tibia by 37%, at the distal femur by 15%, and at L4 by 31% in aged rats (*p* < 0.05 vs. NCD, Table 6). Moreover, HCD and OA effectively improved bone micro-architecture in aged rats (vs. NCD) at all three bone sites. Especially in L4, OA improved Tb.N, Tb.Sp, and Conn.D in aged rats to a comparable level with that of the HCD group (Table 6).

### 3.8. Effect of OA on Renal CYP27B1 and CYP24A1 mRNA and Protein Expression in Aged Female Rats

**Figure 3:**
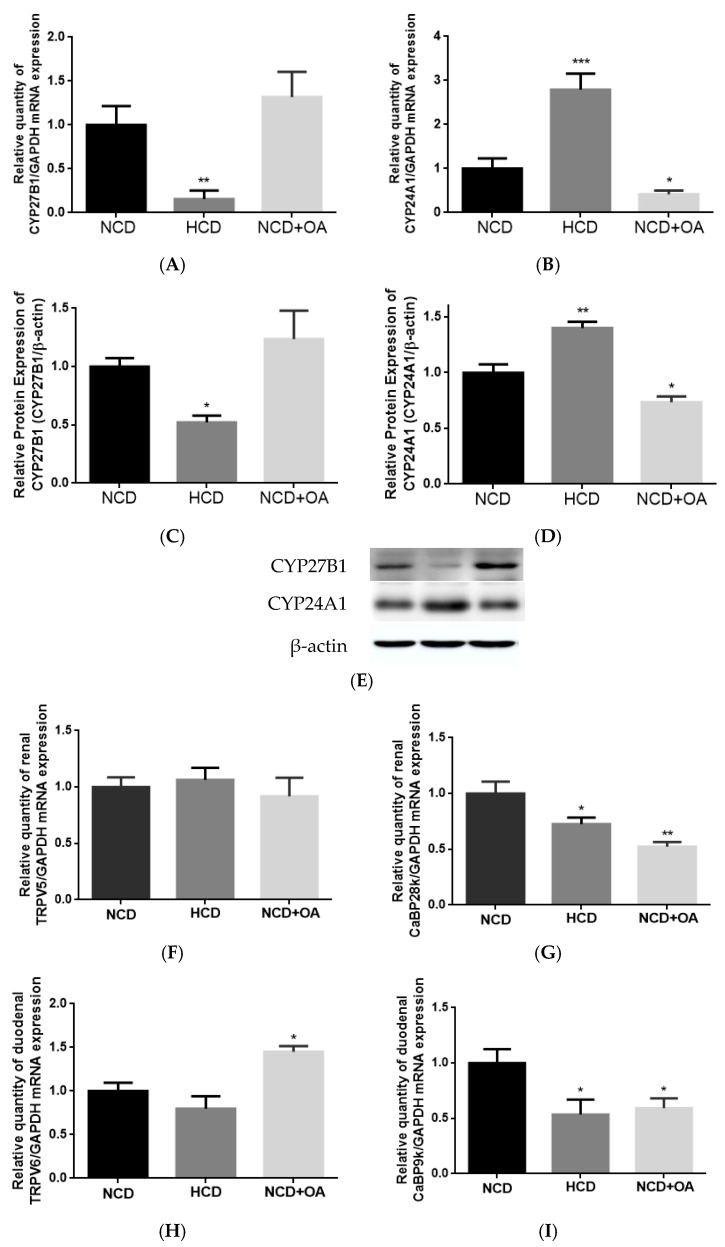
The effect of OA on renal and duodenal mRNA and protein expressions in aged female rats. The expression levels of (**A**) renal CYP27B1 mRNA; (**B**) renal CYP24A1 mRNA; (**C**,**E**) renal CYP27B1 protein; (**D**,**E**) renal CYP24A1 protein; (**F**) renal TRPV5 mRNA; (**G**) renal CaBP28k mRNA; (**H**) duodenal TRPV6 mRNA; and (**I**) duodenal CaBP9k mRNA in aged female rats were studied. Thirteen-month-old female rats were fed with a high calcium diet (HCD, 1.2% calcium, 0.65% phosphorous) or a normal calcium diet (NCD, 0.6% calcium, 0.65% phosphorous) and orally administrated with OA (25 mg/kg/day) or its vehicle treatment for 12 weeks. The mRNA expression level is presented as the ratio of the target gene to GAPDH. Data are presented as mean ± SEM and analyzed by one-way ANOVA followed by Tukey’s multiple comparison tests. * *p* < 0.05, ** *p* < 0.01, and *** *p* < 0.001 vs. NCD.

The effect of HCD and OA treatments on renal CYP27B1 and CYP24A1 mRNA and protein expression in aged female rats are shown in Figure 3. As anticipated, HCD suppressed renal CYP27B1 mRNA and protein expressions (vs. NCD, Figure 3A,C,E) and induced renal CYP24A1 mRNA and protein expressions (vs. NCD, Figure 3B,D,E) in aged rats. OA treatment significantly suppressed the mRNA and protein expressions of renal CYP24A1 (vs. NCD, Figure 3B,D,E) but did not alter CYP27B1 expressions in aged rats.

### 3.9. Effect of OA on Renal and Duodenal mRNA Expression for Calcium Transport in Aged Rats

OA and HCD significantly suppressed renal CaBP28k (vs. NCD, Figure 3G) mRNA expression but did not alter renal TRPV5 (Figure 3F) mRNA expression in aged rats. Both treatments were also found to suppress duodenal CaBP9k in aged rats (vs. NCD, Figure 3I). In contrast, OA treatment, but not HCD, significantly induced duodenal TRPV6 mRNA expression in aged female rats (*p* < 0.05 vs. NCD, Figure 3H).

### 3.10. Effect of OA on CYP27B1 and CYP24A1 mRNA, Protein Expressions and Promoter Activities in HKC-8 Cells

**Figure 4:**
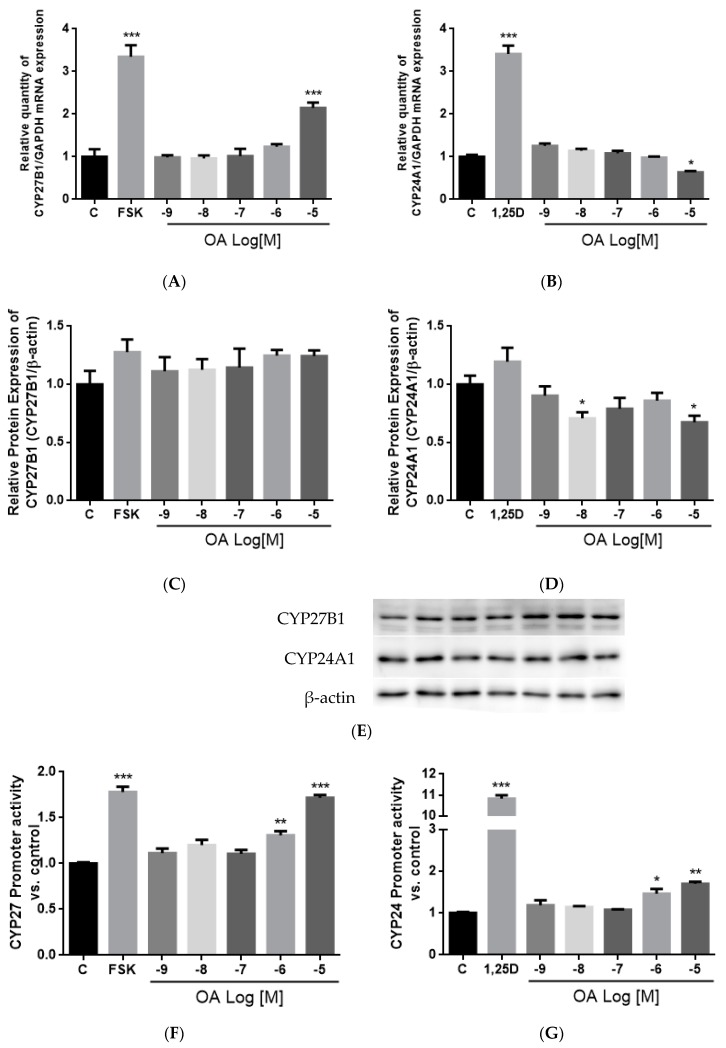
Effects of OA on CYP27B1 and CYP24A1 mRNA, protein expressions, and promoter activities in HKC-8 cells. The expression levels of (**A**) CYP27B1 mRNA; (**B**) CYP24A1 mRNA; (**C**) CYP27B1 protein; (**D**) CYP24A1 protein as well as the promoter activities of (**E**) CYP27B1 and (**F**) CYP24A1 in HKC-8 cells were studied. HKC-8 cells were treated with vehicle (0.1% ethanol), 10^−7^ M PTH (1–34, human), 10^−5^ M Foskolin or 10^−8^ M 1,25(OH)_2_D_3_, and 10^−9^ M–10^−5^ M OA for 24 h. Cells were harvested by Trizol reagent at indicated time for RT-PCR and real-time PCR analysis (**A**,**B**). Relative gene expression was normalized by GAPDH. Total protein was extracted by lysis buffer and separated by SDS-PAGE and immunoblotted with anti-CYP27B1 (**C**,**E**), anti-CYP24A1 (**D**,**E**) antibody and normalized with β-actin expression. Promoter activities (**F**,**G**) were measured by dual luciferase assay and data were normalized against a thymidine kinase (TK) reporter construct. Results are presented as mean ± SEM (*n* = 3) and analyzed by one-way ANOVA followed by Tukey’s multiple comparison test. * *p* < 0.05, ** *p* < 0.01 and *** *p* < 0.001 vs. the control.

To further investigate the effect of OA on CYP27B1 and CYP24A1 expressions, human proximal tubule HKC-8 cells were employed. Following treatment for 24 h, OA at 10^−5^ M significantly induced CYP27B1 mRNA (*p* < 0.001 vs. control, Figure 4A), but not protein, expression in HKC-8 cells (Figure 4C,E). On the other hand, OA significantly suppressed CYP24A1 mRNA expression at 10^−5^ M (*p* < 0.05 vs. control, Figure 4B) and CYP24A1 protein expression at 10^−8^ M and 10^−5^ M (*p* < 0.05 vs. control, Figure 4D,E) in HKC-8 cells. In addition, OA at 10^−6^ M and 10^−5^ M significantly induced CYP27B1 promoter activities in transfected HKC-8 cells by 30% and 71%, respectively (*p* < 0.01, *p* < 0.001, respectively vs. control, Figure 4F). However, OA at 10^−6^ M and 10^−5^ M unexpectedly increased CYP24A1 promoter activities in transfected HKC-8 cells (*p* < 0.05, *p* < 0.01, respectively vs. the control, Figure 4G).

## 4. Discussion

The present study demonstrates that OA could improve estrogen deficiency-induced and age-related bone loss and deterioration of bone properties in mature OVX mice and aged female rats, respectively. The positive action of OA on bone is partially associated with its suppression of urinary calcium loss and increase in serum 1,25(OH)_2_D_3_ levels in OVX mice. The improvement of bone health by OA in aged rats might involve its actions to increase calcium balance. Our results also indicated that OA bestows beneficial effects on bone health, calcium balance and vitamin D metabolism. The mechanism by which OA regulates vitamin D metabolism might be, at least in part, associated with its actions on vitamin D metabolic enzymes, in vivo.

OA increased BMD and improved bone microarchitecture at three bone sites (proximal tibia, distal femur and lumbar vertebra L4) in both OVX mice and aged rats. Our results agree with previous reports in which a quinoxaline derivative of OA was shown to increase the BMD in femurs of OVX mice [20], and 3-months of treatment with OA increased BV/TV in OVX rats [21]. An increase in connectivity density (a crucial structural property of cancellous bone) was shown to improve cancellous bone strength and reduce the risk of fractures by others [48]. Thus, the significant increase in connectivity density at all three sites of OVX mice and aged rats by OA suggests that it might also strengthen cancellous bone. However, the increased BMD and bone microarchitecture may not result in a reduced fracture risk [49,50]. It would be of interest to further investigate whether OA increases biomechanical strength at different bone sites of relevant animal models, especially in a larger number of aged rats, in our future study.

OA behaves like E2, which exerts bone protective actions in an OVX model. E2 as well as OA, at 50 and 100 mg/kg/body weights, was shown to improve BMD and bone properties at three bone sites of OVX mice. However, unlike E2, OA did not suppress the OVX-induced increases in serum OCN and urinary DPD levels in mice. Thus, the bone protective effects exerted by OA in OVX mice might be different from E2 and mediated by distinct mechanisms of actions. In addition, OA increased BMD and improved bone properties in an aged rat model. Such protective actions of OA might be mediated by its reported bone anti-resorption activities [17,18,19,23] and/or the reported stimulatory effects on bone formation [21,24]. In addition, the bone protective actions of OA in vivo might also be mediated indirectly by other hormones, such as 1,25(OH)_2_D_3_ and PTH.

OA mimicked E2 in suppressing urinary calcium loss and inducing renal TRPV5 mRNA expression in OVX mice. However, renal TRPV5 expression could be independently induced by 1,25(OH)_2_D_3_ [51] and estrogen [52] in OVX mice. Since serum 1,25(OH)_2_D_3_ levels were also increased in OVX mice, it is unclear if the induction of renal TRPV5 mRNA by OA is mediated via its E2-like actions or via its actions on 1,25(OH)_2_D_3_. Indeed, unlike E2, OA did not increase duodenal CaBP9K mRNA in OVX mice, suggesting that its actions on Ca transport protein expression might be distinct from estrogen. Using an *in silico* target identification tool [53], OA was predicted to interact with estrogen receptors (ER), among the hundreds of predicted targets for osteoporosis [54]. However, the results of the competitive receptor binding assay from our laboratory indicated that OA did not bind to estrogen receptor α (ERα) or β (ERβ) (Supplementary Materials Figure S1), suggesting that OA did not activate classical estrogen signaling pathway. Thus, the actions of OA in improving calcium re-absorption might be mediated by non-classical estrogen signaling pathways or indirectly via an increase in serum 1,25(OH)_2_D_3_ level.

OA mimicked HCD in improving calcium balance in aged female rats. The improvement in Ca balance by OA was associated with an increase in duodenal TRPV6 mRNA expression in aged female rats. Thus, apart from the direct effects on bone, our results indicated that OA might protect bone through enhancing calcium balance in aged rats, which, in turn, via its actions in regard to increasing calcium entry across brush border membranes [34]. However, duodenal CaBP9K and renal CaBP28K mRNA expression in aged female rats was unexpectedly suppressed by OA in a similar action to that of HCD. The decrease in duodenal CaBP9K and renal CaBP28K by HCD was likely due to the decrease in serum 1,25(OH)_2_D_3_ in aged rats with excess dietary Ca intake [55]. However, as OA did not alter serum 1,25(OH)_2_D_3_ in aged rats, a vitamin D-independent mechanism might be involved in this action. As the activities and protein expression of the calcium transport proteins were not been measured in the present study, future studies will be needed to characterize the effects of OA as well as its mechanism of actions involved in regulation of intestinal and renal Ca transport in aged rats.

Our results are the first to report that OA at 100 mg/kg/day could significantly increase serum 1,25(OH)_2_D_3_ levels and renal CYP27B1 mRNA expression in OVX mice following treatment for 6 weeks. The actions of OA on vitamin D metabolism in OVX mice appeared to be different from those of E2. The present study as well as others [56,57] showed that E2 did not raise serum 1,25(OH)_2_D_3_ levels in OVX mice. In addition, E2 could induce CYP27B1 and suppress CYP24A1 expression in our study using mice and in other studies using avian species [58] models. The mechanisms involved in the alteration of vitamin D metabolism by OA in OVX mice remains to be determined. In contrast, OA at 25 mg/kg/day did not alter serum 1,25(OH)_2_D_3_ levels nor renal CYP27B1 expression in aged female rats following treatment for 3 months. This observation agrees with those reported for aged rats in which the induction of renal CYP27B1 by stimuli, such as PTH, were blunted [59]. In contrast, OA could significantly suppress renal CYP24A1 mRNA and protein expressions in aged rats. In fact, renal CYP24A1 appeared to be more sensitive to the stimuli in aged rats than renal CYP27B1 [2]. Thus, it is possible that OA could alter renal vitamin D metabolism in aged rats via its actions on renal CYP24A1 expression. Recent studies suggest that extra-renal CYP27B1 plays an important role in paracrine and autocrine control of 1,25(OH)_2_D_3_ production [60,61]. As OA significantly improved bone properties and induced duodenum TRPV6 protein expression without altering serum 1,25(OH)_2_D_3_ levels, future study will be needed to study the mechanism of OA on regulating the local production of 1,25(OH)_2_D_3_ in bone and duodenum in aged rats.

OA significantly induced CYP27B1 and mRNA and stimulated CYP27B1 promoter activity in a dose-dependent manner, suggesting that OA regulated CYP27B1 expression transcriptionally. The induction of OA on CYP27B1 mRNA and promoter activity were of similar levels as those induced by PTH and forskolin in HKC-8, as reported by previous studies [62,63]. In addition, OA suppressed mRNA and protein expressions of CYP24A1 in HKC-8 cells, and this result agrees with the action of OA in aged rats. However, a high dose of OA unexpectedly increased CYP24A1 promoter activity in HKC-8 cells. Such unexpected CYP24A1 regulation was reported for the actions of PTH in porcine proximal tubule cell AOK-B50 cells transfected with a full length CYP24A1 promoter [64]. In that study, PTH was shown to work synergistically with 1,25(OH)_2_D_3_ to further induce, rather than inhibit, CYP24A1 promoter activity. The discrepancies between the responses of endogenous CYP24A1 expression and the transfected CYP24A1 promoter activities could be accounted for by the composition of the promoter region in the plasmid constructs. Indeed, the promoter employed in the present study is a partial promoter with a 5′-flanking region and partial exon 1 of CYP24A1 promoter which contains a vitamin D receptor (VDR) responsive region [46,47], and a more distal promoter sequence or 3′ untranslated region of the CYP24A1 promoter might be required for the inhibitory effects of OA.

One limitation of our study was that we only measured the renal CYP27B1 and CYP24A1 expression levels and mRNA expressions of the Ca-transporter in the intestine and kidney. Although we observed beneficial effects of OA on bone, Ca balance, and modulatory effects on vitamin D metabolism, the molecular targets and mechanisms involved in OA regulating Ca and vitamin D metabolism remain unclear. Further studies will be needed to investigate if OA could alter local production of 1,25(OH)_2_D_3_ via its actions on extra-renal CYP27B1 in bone and duodenum in both animal models. Future studies of the actions of OA on intestinal Ca absorption in OVX animal models as well as the proteins and activities of intestinal and renal Ca-transporters would have provided more evidence on the mechanisms involved. 

## 5. Conclusions

This study demonstrates that OA exerts osteoprotective effects via multiple in vivo targets. Apart from the reported effects of OA on bone cells [17,18,19], the present studies show that the protective actions of OA against estrogen deficiency-induced and age-related bone deterioration were, in part, associated with its actions on improving calcium balance and modulating vitamin D metabolism. Since habitual calcium intake is inadequate amongst elderly and postmenopausal women, it is important to have an agent that could improve calcium balance with normal calcium intake. Oral agents that are known to regulate calcium balance and modulate vitamin D metabolism are very limited. The present study demonstrated that OA can improve calcium balance and protect against bone loss associated with estrogen deficiency and aging, which makes OA a potential low-cost, orally administrated drug candidate for the management of osteoporosis.

## Figures and Tables

**Table 1:** Primer sequences.

Gene	Primer Sequence (5′-3′)	
Mouse		
GAPDH	F: CAGAACATCATCCCTGCATC	R: CTGCTTCACCACCTTCTTGA
CYP27B1	F: GCATCACTTAACCCACTTCC	R: CGGGAAAGCTCATAGAGTGT
CYP24A1	F: AAGAGATTCGGGCTCCTTCA	R: GCAGGGCTTGACTGATTTGA
TRPV5	F: GAAACTTCTCAATTGGTGGGTCAG	R: TTTGCCGGAAGTCACAGTT
Rat		
GAPDH	F: GTGAGGTGACCGCATCTTCT	R: CTTGCCGTGGGTAGAGTCAT
CYP27B1	F: CCATCGAGTCCAACTGCCTT	R: AGGGTCGGCCACATAAACTG
CYP24A1	F: CTCGGACCCTTGACAAACCA	R: CGATGCCGAATGGGAGATGA
TRPV6	F: ATCCGCCGCTATGCACA	R: AGTTTTTTCTCCTGAGTCTTTTTCCA
Human		
GAPDH	F: TTGCAACCGGGAAGGAAATG	R: CGCCCAATACGACCAAATCA
CYP27B1	F: CTGCGGAAGGCGAAGAATGG	R: TTGTTCAGGGTTCCGGCGTA
CYP24A1	F: CAAACCGTGGAAGGCCTATC	R: AGTCTTCCCCTTCCAGGATCA

CYP27B1, 25-hydroxyvitamin D 1-α-hydroxylase; CYP24A1, 25-hydroxyvitamin D 24-hydroxylase; GAPDH, glyceraldehyde-3-phosphate dehydrogenase.; TRPV5/6, Transient Receptor Potential channel, Vanilloid subfamily member 5/6.

**Table 2:** Effect of oleanolic acid (OA) on body weight change, uterus index, biochemical parameters, and bone calcium content in ovariectomised (OVX) mice.

	Sham	OVX	E2	OAL	OAH
Body weight and uterus index					
Weight change, %	1.3 ± 0.7	7.3 ± 1.3 ^^^	−6.6 ± 1.1 ***	−3.9 ± 1.0 ***	−7.4 ± 1.4 ***
Uterus index, mg/g	0.31 ± 0.03	0.09 ± 0.02 ^^^	0.70 ± 0.08 ***	0.12 ± 0.02	0.16 ± 0.02
Serum chemistry					
s-Ca, mg/dL	8.7 ± 0.2	8.9 ± 0.2	9.2 ± 0.2	9.2 ± 0.3	9.1 ± 0.3
s-P, mg/dL	7.6 ± 0.4	7.6 ± 0.6	6.7 ± 0.6	6.9 ± 0.8	6.5 ± 0.5
s-OCN, ng/mL	77.2 ± 1.4	86.6 ± 2.9 ^	72.0 ± 3.1 **	93.5 ± 4.0	93.6 ± 4.8
s-1,25D, pg/mL	63.3 ± 5.1	74.6 ± 2.5	58.8 ± 6.9	83.9 ± 8.5	98.7 ± 7.2 *
Urine chemistry					
u-Ca/Cr, mg/mg	0.21 ± 0.03	0.42 ± 0.04 ^^^	0.29 ± 0.02 *	0.16 ± 0.02 ***	0.14 ± 0.01 ***
u-P/Cr, mg/mg	7.3 ± 0.4	7.6 ± 0.6	6.5 ± 0.2	6.5 ± 0.5	6.6 ± 0.3
u-DPD, nmol/mmol	9.4 ± 0.5	14.4 ± 1.2 ^^^	8.2 ± 0.6 ***	11.7 ± 0.8	14.8 ± 0.5
Bone calcium content					
Bone ash weight, mg	19.7 ± 0.4	18.6 ± 0.3	21.1 ± 0.4 *	20.8 ± 0.6 *	21.0 ± 0.8 *
Ca/Ash bone, μg/mg	377.4 ± 1.6	372. 3 ± 2.8	387.7 ± 3.9 *	387.0 ± 2.7 *	387.3 ± 3.3 *

Four-month-old ovariectomized (OVX) or sham-operated (Sham) C57BL/6J mice were pair-fed with a phytoestrogen-free AIN-93M diet and treated with vehicle (Sham or OVX), E2 (200 μg/kg/day), OA low dose (OAL, 50 mg/kg/day) or OA high dose (OAH, 100 mg/kg/day) for 6 weeks. Body weight (% of change) from baseline to 6 weeks.1,25D, 1,25(OH)_2_D_3_; s, serum; u, urine; Ca, calcium; P, phosphorus; Cr, creatinine. Urinary calcium, phosphorus and urinary deoxypyridinoline (DPD) levels were expressed as urinary calcium, phosphorus or DPD to creatinine ratio. Data are presented as mean ± standard error of mean (SEM) and analyzed by one-way ANOVA followed by Tukey’s multiple comparison tests. ^ *p* < 0.05 and ^^^ *p* < 0.001 vs. Sham; * *p* < 0.05, ** *p* < 0.01, and *** *p* < 0.001 vs. OVX.

**Table 3:** Effect of OA on bone mineral density and bone microarchitecture at proximal tibia, distal femur, and lumbar vertebra in OVX mice analyzed by micro-CT.

	Sham	OVX	E2	OAL	OAH
Proximal tibia					
BMD, mg HA/cm^3^	207.2 ± 7.7	116.7 ± 6.5 ^^^	249.4 ± 7.5 ***	170.5 ± 6.8 ***	175.4 ± 4.7 ***
BV/TV, %	23.3 ± 1.1	11.5 ± 1.0 ^^^	27.9 ± 1.4 ***	18.0 ± 0.8 ***	19.0 ± 0.7 ***
Tb.N, mm^−1^	4.51 ± 0.24	3.08 ± 0.37	5.38 ± 0.29 ***	3.76 ± 0.15	3.74 ± 0.08
Tb.Th, μm	51.2 ± 0.8	42.9 ± 1.6 ^^^	54.0 ± 1.2 ***	48.7 ± 0.8 **	50.7 ± 1.1 ***
Tb.Sp, μm	175.4 ± 11.2	312.2 ± 34.7 ^^^	126.5 ± 17.3 ***	225.4 ± 11.1 *	217.6 ± 6.5 *
Conn.D, mm^3^	196.1 ± 15.9	105.6 ± 8.3 ^^	237.2 ± 27.7 ***	159.6 ± 10.0 ***	140.4 ± 8.9 *
Distal femur					
BMD, mg HA/cm^3^	198.4 ± 6.6	130.9 ± 9.1 ^^^	246.8 ± 8.4 ***	185.9 ± 4.3 ***	195.6 ± 7.2 ***
BV/TV, %	16.6 ± 0.9	12.0 ± 1.0 ^^	25.5 ± 1.3 ***	18.5 ± 0.8 ***	20.7 ± 1.1 ***
Tb.N, mm^−1^	3.50 ± 0.08	2.79 ± 0.18 ^	4.24 ± 0.18 ***	3.57 ± 0.14 *	3.81 ± 0.21 ***
Tb.Th, μm	50.3 ± 1.5	41.5 ± 1.4 ^^^	59.4 ± 2.1 ***	51.9 ± 1.4 ***	54.2 ± 0.4 ***
Tb.Sp, μm	239.7 ± 8.0	329.8 ± 27.6 ^^	176.3 ± 11.0 ***	231.6 ± 10.9 **	214.2 ± 15.9 ***
Conn.D, mm^3^	141.8 ± 11.5	97.4 ± 7.9 ^	141.6 ± 10.8 *	144.5 ± 8.5 *	149.2 ± 10.7 **
Lumbar vertebra					
BMD, mg HA/cm^3^	231.8 ± 11.0	183.1 ± 7.1 ^^^	297.0 ± 15.3 ***	219.0 ± 8.0 **	213.2 ± 4.7 *
BV/TV, %	29.4 ± 1.1	21.1 ± 0.6 ^^^	37.0 ± 1.7 ***	25.6 ± 1.3 **	25.1 ± 0.8 *
Tb.N, mm^−1^	4.62 ± 0.12	3.82 ± 0.08 ^^^	4.89 ± 0.11 ***	4.26 ± 0.13 *	4.33 ± 0.12 **
Tb.Th, μm	63.5 ± 1.0	58.3 ± 1.9	75.3 ± 2.0 ***	59.3 ± 1.5	57.0 ± 1.0
Tb.Sp, μm	153.8 ± 6.3	208.8 ± 5.7 ^^^	130.0 ± 6.3 ***	176.1 ± 7.7 **	172.9 ± 5.2 **
Conn.D, mm^3^	141.1 ± 7.1	100.2 ± 4.9 ^^^	139.7 ± 4.3 ***	140.8 ± 6.5 ***	140.3 ± 6.6 ***

Four-month-old ovariectomized (OVX) or sham-operated (Sham) C57BL/6J mice were pair-fed with phytoestrogen-free AIN-93M diet and treated with vehicle (Sham or OVX), E2 (200 μg/kg/day), OA low dose (OAL, 50 mg/kg/day) or OA high dose (OAH, 100 mg/kg/day) for 6 weeks. Bone mineral density (BMD) and bone microarchitecture parameters were measured by microCT: bone volume/total volume (BV/TV), trabecular number (Tb.N), trabecular thickness (Tb.Th), trabecular separation (Tb.Sp), and connectivity density (Conn.D). Data are presented as mean ± SEM and analyzed by one-way ANOVA followed by Tukey’s multiple comparison tests. ^ *p* < 0.05, ^^ *p* < 0.01, ^^^ *p* < 0.001 vs. Sham; and * *p* < 0.05, ** *p* < 0.01, *** *p* < 0.001 vs. OVX.

**Table 4:** Effects of OA on body weight and biochemical parameters in aged female rats.

	NCD	HCD	NCD + OA
Body weight			
Weight change, %	−1.3 ± 0.4	−0.4 ± 0.7	0.2 ± 1.6
Serum chemistry			
s-Ca, mg/dL	11.5 ± 0.4	11.5 ± 0.2	11.9 ± 0.5
s-P,mg/dL	5.2 ± 0.4	5.9 ± 0.2	4.9 ± 0.8
s-1,25D, pg/mL	17.5 ± 2.5	3.5 ± 0.7 ***	13.4 ± 2.9
s-PTH, pg/mL	213.8 ± 40.9	229.2 ± 49.4	138.2 ± 58.0
Urine chemistry			
u-Ca/Cr, mg/mg	0.30 ± 0.05	0.38 ± 0.04	0.16 ± 0.03 *
u-P/Cr, mg/mg	2.65 ± 0.43	0.12 ± 0.07 ***	2.11 ± 0.24
Bone calcium content			
Bone ash weight, mg	306.1 ± 8.5	351.6 ± 6.1 **	318.2 ± 8.0
Ca/Ash bone, μg/mg	391.8 ± 2.2	403.1 ± 3.6 *	402.8 ± 3.3 *

Thirteen-month-old female rats were fed with a high calcium diet (HCD, 1.2% calcium, 0.65% phosphorous) or a normal calcium diet (NCD, 0.6% calcium, 0.65% phosphorous) and orally administrated with OA (25 mg/kg/day) or its vehicle treatment for 12 weeks. Body weight is presented as percentage of change from baseline to 12 weeks. 1,25D, 1,25(OH)_2_D_3_; s, serum; u, urine; Ca, calcium; P, phosphorus; Cr, creatinine. Urinary calcium and phosphorus levels are expressed as urinary calcium or phosphorus to creatinine ratio. Data is presented as mean ± SEM and analyzed by one-way ANOVA followed by Tukey’s multiple comparison tests. * *p* < 0.05, ** *p* < 0.01, *** *p* < 0.001 vs. NCD.

**Table 5:** Effects of OA on Ca balance in aged female rats.

	NCD	HCD	NCD + OA
Ca intake, mg/day	92.4 ± 3.9	182.1 ± 11.2	94.2 ± 4.7
Urine Ca, mg/day	1.8 ± 0.7	5.7 ± 0.6 ***	1.4 ± 0.2
Fecal Ca, mg/day	80.2 ± 5.6	158.1 ± 19.4 **	68.5 ± 5.0
Ca absorption rate, %	11.2 ± 4.7	12.2 ± 10.8	23.9 ± 5.9
Net Ca balance, mg/day	12.3 ± 2.2	26.7 ± 4.9 *	24.9 ± 3.0 *

Thirteen-month-old female rats were fed with a high calcium diet (HCD, 1.2% calcium, 0.65% phosphorous) or a normal calcium diet (NCD, 0.6% calcium, 0.65% phosphorous) and orally administrated with OA (25 mg/kg/day) or its vehicle treatment for 12 weeks. Ca absorption rate (%) = (Ca intake − fecal Ca)/Ca intake × 100; Net Ca balance = Ca intake − (urinary Ca − fecal Ca). Data is presented as mean ± SEM and analyzed by one-way ANOVA followed by Tukey’s multiple comparison tests. * *p* < 0.05, ** *p* < 0.01, *** *p* < 0.001 vs. NCD.

**Table 6:** Effect of OA on bone mineral density and bone microarchitecture at the proximal tibia, distal femur, and lumbar vertebra in aged female rats, analyzed by micro-CT.

	NCD	HCD	NCD + OA
Proximal tibia			
BMD, mg HA/cm^3^	225.7 ± 21.1	330.1 ± 18.0 **	312.0 ± 16.9 *
BV/TV, %	34.9 ± 3.5	50.0 ± 3.3 *	46.1 ± 2.8 *
Tb.N, mm^−1^	3.19 ± 0.13	3.76 ± 0.10 **	3.61 ± 0.08 *
Tb.Th, μm	107.0 ± 7.3	136.6 ± 7.4 *	122.9 ± 6.2
Tb.Sp, μm	205.2 ± 20.3	134.6 ± 11.4 **	148.4 ± 10.2 *
Conn.D, mm^3^	31.4 ± 2.1	46.9 ± 2.1 **	43.2 ± 2.2 **
Distal femur			
BMD, mg HA/cm^3^	282.1 ± 9.8	391.9 ± 19.7 **	324.3 ± 10.6 *
BV/TV, %	35.9 ± 3.4	57.7 ± 4.2 **	47.5 ± 2.1 *
Tb.N, mm^−1^	2.95 ± 0.08	3.32 ± 0.04 *	3.31 ± 0.07 *
Tb.Th, μm	132.5 ± 4.6	184.3 ± 12.4 *	147.8 ± 5.1
Tb.Sp, μm	200.9 ± 0.4	128.3 ± 12.4 ***	156.2 ± 9.3 *
Conn.D, mm^3^	23.7 ± 1.8	30.1 ± 1.6 *	31.6 ± 1.4 *
Lumbar vertebra			
BMD, mg HA/cm^3^	286.7 ± 13.0	407.7 ± 18.5 ***	378.1 ± 18.0 *
BV/TV, %	31.2 ± 2.2	49.7 ± 2.9 **	46.1 ± 2.8 *
Tb.N, mm^−1^	3.02 ± 0.08	3.59 ± 0.08 ***	3.51 ± 0.07 ***
Tb.Th, μm	102.9 ± 5.8	139.6 ± 10.4 *	123.8 ± 9.3
Tb.Sp, μm	229.2 ± 12.0	139.5 ± 7.0 ***	155.5 ± 8.0 ***
Conn.D, mm^3^	30.4 ± 1.1	39.3 ± 1.5 **	40.8 ± 1.0 ***

Thirteen-month-old female rats were fed with a high calcium diet (HCD, 1.2% calcium, 0.65% phosphorous) or a normal calcium diet (NCD, 0.6% calcium, 0.65% phosphorous) and orally administrated with OA (25 mg/kg/day) or its vehicle treatment for 12 weeks. Bone mineral density (BMD) and bone microarchitecture parameters were measured by microCT: bone volume/total volume (BV/TV), trabecular number (Tb.N), trabecular thickness (Tb.Th), trabecular separation (Tb.Sp), and connectivity density (Conn.D). Data is presented as mean ± SEM and analyzed by one-way ANOVA followed by Tukey’s multiple comparison tests. * *p* < 0.05, ** *p* < 0.01 and *** *p* < 0.001 vs. NCD.

## Supplementary Materials

The following are available online at http://www.mdpi.com/2072-6643/10/2/247/s1, Figure S1: Competitive ligand binding assays of oleanolic acid for binding to ERα (A) and ERβ (B).

## References

[1] Drake M.T., Clarke B.L., Lewiecki E.M. (2015). The Pathophysiology and Treatment of Osteoporosis. Clin. Ther..

[2] Veldurthy V., Wei R., Oz L., Dhawan P., Jeon Y.H., Christakos S. (2016). Vitamin D, calcium homeostasis and aging. Bone Res..

[3] Tella S.H., Gallagher J.C. (2014). Prevention and treatment of postmenopausal osteoporosis. J. Steroid Biochem. Mol. Biol..

[4] Nordin B. (1997). Calcium and osteoporosis. Nutrition.

[5] Halloran B.P., Portale A.A., Feldman D., Pike J.W., Glorieux F. (2005). Vitamin D metabolism and aging. Vitamin D.

[6] Riggs B.L. (2003). Role of the vitamin D-endocrine system in the pathophysiology of postmenopausal osteoporosis. J. Cell. Biochem..

[7] Tang B.M., Eslick G.D., Nowson C., Smith C., Bensoussan A. (2007). Use of calcium or calcium in combination with vitamin D supplementation to prevent fractures and bone loss in people aged 50 years and older: A meta-analysis. Lancet.

[8] Bolland M.J., Barber P.A., Doughty R.N., Mason B., Horne A., Ames R., Gamble G.D., Grey A., Reid I.R. (2008). Vascular events in healthy older women receiving calcium supplementation: Randomised controlled trial. BMJ.

[9] Chung M., Tang A.M., Fu Z., Wang D.D., Newberry S.J. (2016). Calcium Intake and Cardiovascular Disease Risk: An Updated Systematic Review and Meta-analysis. Ann. Intern. Med..

[10] Oudshoorn C., van der Cammen T.J., McMurdo M.E., van Leeuwen J.P., Colin E.M. (2009). Ageing and vitamin D deficiency: Effects on calcium homeostasis and considerations for vitamin D supplementation. Br. J. Nutr..

[11] Warensjo E., Byberg L., Melhus H., Gedeborg R., Mallmin H., Wolk A., Michaelsson K. (2011). Dietary calcium intake and risk of fracture and osteoporosis: Prospective longitudinal cohort study. BMJ.

[12] Avenell A., Mak J.C., O’Connell D. (2014). Vitamin D and vitamin D analogues for preventing fractures in post-menopausal women and older men. Cochrane Database Syst. Rev..

[13] Bolland M.J., Leung W., Tai V., Bastin S., Gamble G.D., Grey A., Reid I.R. (2015). Calcium intake and risk of fracture: Systematic review. BMJ.

[14] Zhao J.G., Zeng X.T., Wang J., Liu L. (2017). Association Between Calcium or Vitamin D Supplementation and Fracture Incidence in Community-Dwelling Older Adults: A Systematic Review and Meta-analysis. JAMA.

[15] Pollier J., Goossens A. (2012). Oleanolic acid. Phytochemistry.

[16] Fukushima E.O., Seki H., Ohyama K., Ono E., Umemoto N., Mizutani M., Saito K., Muranaka T. (2011). CYP716A subfamily members are multifunctional oxidases in triterpenoid biosynthesis. Plant Cell Physiol..

[17] Li J.-X., Hareyama T., Tezuka Y., Zhang Y., Miyahara T., Kadota S. (2005). Five new oleanolic acid glycosides from Achyranthes bidentata with inhibitory activity on osteoclast formation. Planta Med..

[18] Zhang Y., Li J., Zhao J., Wang S., Pan Y., Tanaka K., Kadota S. (2005). Synthesis and activity of oleanolic acid derivatives, a novel class of inhibitors of osteoclast formation. Bioorg. Med. Chem. Lett..

[19] Li J.F., Zhao Y., Cai M.M., Li X.F., Li J.X. (2009). Synthesis and evaluation of a novel series of heterocyclic oleanolic acid derivatives with anti-osteoclast formation activity. Eur. J. Med. Chem..

[20] Zhao Y., Huai Y., Jin J., Geng M., Li J.-X. (2011). Quinoxaline derivative of oleanolic acid inhibits osteoclastic bone resorption and prevents ovariectomy-induced bone loss. Menopause.

[21] Bian Q., Liu S.F., Huang J.H., Yang Z., Tang D.Z., Zhou Q., Ning Y., Zhao Y.J., Lu S., Shen Z.Y. (2012). Oleanolic acid exerts an osteoprotective effect in ovariectomy-induced osteoporotic rats and stimulates the osteoblastic differentiation of bone mesenchymal stem cells in vitro. Menopause.

[22] Kim J.Y., Cheon Y.H., Oh H.M., Rho M.C., Erkhembaatar M., Kim M.S., Lee C.H., Kim J.J., Choi M.K., Yoon K.H. (2014). Oleanolic acid acetate inhibits osteoclast differentiation by downregulating PLCgamma2-Ca(2+)-NFATc1 signaling, and suppresses bone loss in mice. Bone.

[23] Xu D., Lyu Y., Chen X., Zhu X., Feng J., Xu Y. (2016). Fructus Ligustri Lucidi ethanol extract inhibits osteoclastogenesis in RAW264.7 cells via the RANKL signaling pathway. Mol. Med. Rep..

[24] Shu B., Zhao Y., Wang Y., Wang G., Shang X., Britt M., Olmedo M., Chelly M., Morandi M.M., Barton S. (2017). Oleanolic Acid Enhances Mesenchymal Stromal Cell Osteogenic Potential by Inhibition of Notch Signaling. Sci. Rep..

[25] Zhang Y., Lai W.P., Leung P.C., Wu C.F., Yao X.S., Wong M.S. (2006). Effects of Fructus Ligustri Lucidi extract on bone turnover and calcium balance in ovariectomized rats. Biol. Pharm. Bull..

[26] China Pharmacopoeia Committee (2010). Chinese Pharmacopoeia.

[27] Zhang Y., Lai W.P., Leung P.C., Che C.T., Wong M.S. (2008). Improvement of Ca balance by Fructus Ligustri Lucidi extract in aged female rats. Osteoporos. Int..

[28] Zhang Y., Dong X.L., Leung P.C., Che C.T., Wong M.S. (2008). Fructus ligustri lucidi extract improves calcium balance and modulates the calciotropic hormone level and vitamin D-dependent gene expression in aged ovariectomized rats. Menopause.

[29] Zhang Y., Leung P.C., Che C.T., Chow H.K., Wu C.F., Wong M.S. (2008). Improvement of bone properties and enhancement of mineralization by ethanol extract of Fructus Ligustri Lucidi. Br. J. Nutr..

[30] Dong X.L., Zhang Y., Favus M.J., Che C.T., Wong M.S. (2010). Ethanol extract of Fructus Ligustri Lucidi increases circulating 1,25-dihydroxyvitamin D3 by inducing renal 25-hydroxyvitamin D-1alpha hydroxylase activity. Menopause.

[31] Dong X.L., Zhao M., Wong K.K., Che C.T., Wong M.S. (2012). Improvement of calcium balance by Fructus Ligustri Lucidi extract in mature female rats was associated with the induction of serum parathyroid hormone levels. Br. J. Nutr..

[32] Feng X., Lyu Y., Wu Z., Fang Y., Xu H., Zhao P., Xu Y., Feng H. (2014). Fructus ligustri lucidi ethanol extract improves bone mineral density and properties through modulating calcium absorption-related gene expression in kidney and duodenum of growing rats. Calcif. Tissue Int..

[33] Lyu Y., Feng X., Zhao P., Wu Z., Xu H., Fang Y., Hou Y., Denney L., Xu Y., Feng H. (2014). Fructus Ligustri Lucidi (FLL) ethanol extract increases bone mineral density and improves bone properties in growing female rats. J. Bone Miner. Metab..

[34] Fleet J.C., Schoch R.D. (2010). Molecular mechanisms for regulation of intestinal calcium absorption by vitamin D and other factors. Crit. Rev. Clin. Lab. Sci..

[35] Murayama A., Takeyama K., Kitanaka S., Kodera Y., Kawaguchi Y., Hosoya T., Kato S. (1999). Positive and negative regulations of the renal 25-hydroxyvitamin D_3_ 1alpha-hydroxylase gene by parathyroid hormone, calcitonin, and 1alpha,25(OH)2D_3_ in intact animals. Endocrinology.

[36] Takeyama K., Kitanaka S., Sato T., Kobori M., Yanagisawa J., Kato S. (1997). 25-Hydroxyvitamin D_3_ 1alpha-hydroxylase and vitamin D synthesis. Science.

[37] Zierold C., Mings J.A., DeLuca H.F. (2003). Regulation of 25-hydroxyvitamin D_3_-24-hydroxylase mRNA by 1,25-dihydroxyvitamin D_3_ and parathyroid hormone. J. Cell. Biochem..

[38] Wang Y., Zhu J., DeLuca H.F. (2015). The vitamin D receptor in the proximal renal tubule is a key regulator of serum 1alpha,25-dihydroxyvitamin D(3). Am. J. Physiol. Endocrinol. Metab..

[39] Ranch D., Zhang M.Y., Portale A.A., Perwad F. (2011). Fibroblast growth factor 23 regulates renal 1,25-dihydroxyvitamin D and phosphate metabolism via the MAP kinase signaling pathway in Hyp mice. J. Bone Miner. Res..

[40] Wong M., Sriussadaporn S., Tembe V.A., Favus M.J. (1997). Insulin-like growth factor I increases renal 1,25 (OH) 2D3 biosynthesis during low-P diet in adult rats. Am. J. Physiol..

[41] Wong M.S., Tembe V.A., Favus M.J. (2000). Insulin-like growth factor-I stimulates renal 1,25-dihydroxycholecalciferol synthesis in old rats fed a low calcium diet. J. Nutr..

[42] Bikle D.D. (2014). Vitamin D metabolism, mechanism of action, and clinical applications. Chem. Biol..

[43] Shimada T., Hasegawa H., Yamazaki Y., Muto T., Hino R., Takeuchi Y., Fujita T., Nakahara K., Fukumoto S., Yamashita T. (2004). FGF-23 is a potent regulator of vitamin D metabolism and phosphate homeostasis. J. Bone Miner. Res..

[44] Racusen L.C., Monteil C., Sgrignoli A., Lucskay M., Marouillat S., Rhim J.G., Morin J.-P. (1997). Cell lines with extended in vitro growth potential from human renal proximal tubule: Characterization, response to inducers, and comparison with established cell lines. J. Lab. Clin. Med..

[45] Chanakul A., Zhang M.Y., Louw A., Armbrecht H.J., Miller W.L., Portale A.A., Perwad F. (2013). FGF-23 regulates CYP27B1 transcription in the kidney and in extra-renal tissues. PLoS ONE.

[46] Kerry D.M., Dwivedi P.P., Hahn C.N., Morris H.A., Omdahl J.L., May B.K. (1996). Transcriptional synergism between vitamin D-responsive elements in the rat 25-hydroxyvitamin D_3_ 24-hydroxylase (CYP24) promoter. J. Biol. Chem..

[47] Kemmis C.M., Salvador S.M., Smith K.M., Welsh J. (2006). Human mammary epithelial cells express CYP27B1 and are growth inhibited by 25-hydroxyvitamin D-3, the major circulating form of vitamin D-3. J. Nutr..

[48] Srivastava K., Khan K., Tyagi A.M., Khan M.P., Yadav D.K., Trivedi R., Maurya R., Singh D., Chattopadhyay N. (2013). Greater Skeletal Gains in Ovary Intact Rats at Maturity Are Achieved by Supplementing a Standardized Extract of Butea monosperma Stem Bark that Confers Better Bone Conserving Effect following Ovariectomy and Concurrent Treatment Withdrawal. Evid. Based Complement. Altern. Med..

[49] Haguenauer D., Welch V., Shea B., Tugwell P., Adachi J.D., Wells G. (2000). Fluoride for the treatment of postmenopausal osteoporotic fractures: A meta-analysis. Osteoporos. Int..

[50] Henriksen K., Byrjalsen I., Andersen J.R., Bihlet A.R., Russo L.A., Alexandersen P., Valter I., Qvist P., Lau E., Riis B.J. (2016). A randomized, double-blind, multicenter, placebo-controlled study to evaluate the efficacy and safety of oral salmon calcitonin in the treatment of osteoporosis in postmenopausal women taking calcium and vitamin D. Bone.

[51] Hoenderop J.G., Dardenne O., Van Abel M., Van Der Kemp A.W., Van Os C.H., St-Arnaud R., Bindels R.J. (2002). Modulation of renal Ca^2+^ transport protein genes by dietary Ca^2+^ and 1,25-dihydroxyvitamin D_3_ in 25-hydroxyvitamin D_3_-1alpha-hydroxylase knockout mice. FASEB J..

[52] Van Abel M., Hoenderop J.G., Dardenne O., St Arnaud R., Van Os C.H., Van Leeuwen H.J., Bindels R.J. (2002). 1,25-dihydroxyvitamin D(3)-independent stimulatory effect of estrogen on the expression of ECaC1 in the kidney. J. Am. Soc. Nephrol..

[53] Wang J., Shan A., Liu T., Zhang C., Zhang Z. (2012). In vitro immunomodulatory effects of an oleanolic acid-enriched extract of *Ligustrum lucidum* fruit (*Ligustrum lucidum* supercritical CO_2_ extract) on piglet immunocytes. Int. Immunopharmacol..

[54] Cao S., Qiu Z., Wong M.S. (2017). The Hong Kong Polytechnic Universtiy, Hong Kong, China.

[55] Armbrecht H.J., Boltz M.A., Bruns M.E. (2003). Effect of age and dietary calcium on intestinal calbindin D-9k expression in the rat. Arch. Biochem. Biophys..

[56] Colin E.M., Van Den Bemd G.J., Van Aken M., Christakos S., De Jonge H.R., Deluca H.F., Prahl J.M., Birkenhager J.C., Buurman C.J., Pols H.A. (1999). Evidence for involvement of 17beta-estradiol in intestinal calcium absorption independent of 1,25-dihydroxyvitamin D_3_ level in the Rat. J. Bone Miner. Res..

[57] Shen V., Dempster D.W., Birchman R., Xu R., Lindsay R. (1993). Loss of cancellous bone mass and connectivity in ovariectomized rats can be restored by combined treatment with parathyroid hormone and estradiol. J. Clin. Investig..

[58] Tanaka Y., Castillo L., DeLuca H.F. (1976). Control of renal vitamin D hydroxylases in birds by sex hormones. Proc. Natl. Acad. Sci. USA.

[59] Armbrecht H.J., Boltz M.A., Ritter C.S., Brown A.J. (2007). Parathyroid hormone stimulation of the renal 25-hydroxyvitamin D-1alpha-hydroxylase—Effect of age and free radicals. J. Steroid Biochem. Mol. Biol..

[60] Anderson P.H., O’Loughlin P.D., May B.K., Morris H.A. (2005). Modulation of CYP27B1 and CYP24 mRNA expression in bone is independent of circulating 1,25(OH)2D_3_ levels. Bone.

[61] Bikle D.D. (2016). Extraskeletal actions of vitamin D. Ann. N. Y. Acad. Sci..

[62] Brenza H.L., Kimmel-Jehan C., Jehan F., Shinki T., Wakino S., Anazawa H., Suda T., DeLuca H.F. (1998). Parathyroid hormone activation of the 25-hydroxyvitamin D_3_-1alpha-hydroxylase gene promoter. Proc. Natl. Acad. Sci. USA.

[63] Brenza H.L., DeLuca H.F. (2000). Regulation of 25-hydroxyvitamin D_3_ 1α-hydroxylase gene expression by parathyroid hormone and 1,25-dihydroxyvitamin D_3_. Arch. Biochem. Biophys..

[64] Zierold C., Reinholz G.G., Mings J.A., Prahl J.M., DeLuca H.F. (2000). Regulation of the procine 1,25-dihydroxyvitamin D_3_-24-hydroxylase (CYP24) by 1,25-dihydroxyvitamin D_3_ and parathyroid hormone in AOK-B50 cells. Arch. Biochem. Biophys..

