# Supplementary material for: Oleanolic Acid Exerts Osteoprotective Effects and Modulates Vitamin D Metabolism
Source: Nutrients. 2018 Feb 22;10(2):247. doi: 10.3390/nu10020247 (PMC5852823; doi:10.3390/nu10020247)
Supplement: Supplementary file 1 [file nutrients-10-00247-s001.pdf]

Supplementary file

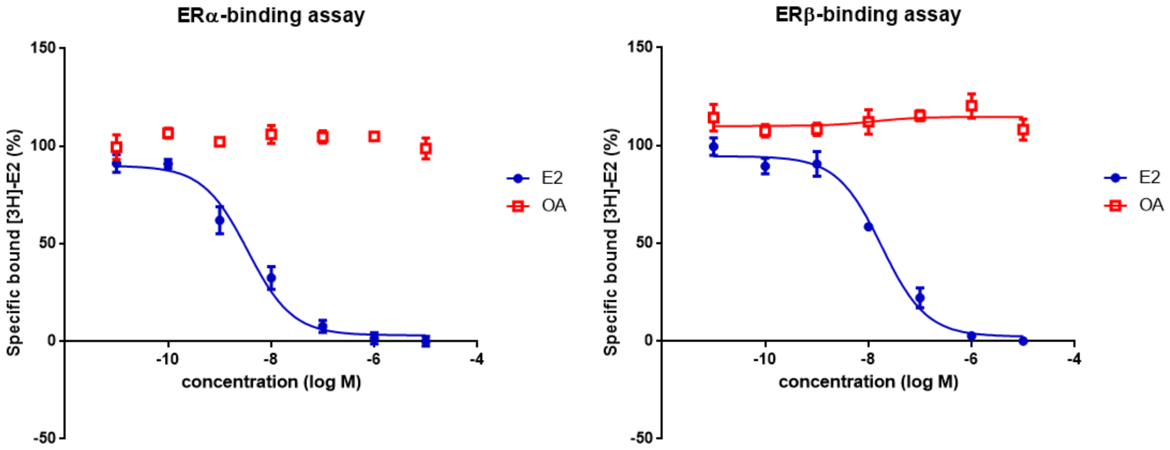

(A) **(B)**

Figure S1. Competitive ligand binding assays of oleanolic acid for binding to ER*α* (A) and ERβ (B). A concentration gradient of OA (10^{-11}M to 10^{-5}M) or non-radiolabeled 17β-oestradiol (10^{-11}M to 10^{-5}M) was co-incubated with and 10^{-8}M [^{3}H]-17β-oestradiol to determine the displacement of specific [^{3}H]-17β-oestradiol binding to human recombinant full length ER*α* and ERβ proteins. Data were presented as percentage specific bound to [^{3}H]-17β-oestradiol. The figures were obtained from two independent experiments (n=6).
